# Load-displacement experimental data from shear loading of hybrid GFRP-graphite filler using a complex Arcan fixture

**DOI:** 10.1016/j.dib.2024.110139

**Published:** 2024-02-03

**Authors:** Ariyana Dwiputra Nugraha, Daffa Alandro, Alvin Dio Nugroho, Eko Supriyanto, Fefria Tanbar, Muhammad Akhsin Muflikhun

**Affiliations:** a PLN Research Institute, Jakarta, Indonesia; b Mechanical and Industrial Engineering Department, Gadjah Mada University, Indonesia

**Keywords:** Arcan fixture, Shear test, Hybrid composite, Material properties

## Abstract

This paper presents a dataset of load-displacement obtained from shear loading tests on pure GFRP laminates and hybrid GFRP-graphite filler laminates. The specimens were cut according to the ASTM D 7078 standard, and the width and thickness of the notch area were measured at least three times. Shear loading was applied at a rate of 2 mm/min, and data were recorded from unloading until specimen failure. The data provides information on the maximum load and unique behavior of GFRP laminates. Based on the obtained load, the shear stress (MPa) unit can be calculated. This data can serve as a basis for researchers and engineers working with GFRP laminates and hybrid GFRP-graphite filler laminates.

Specifications Table

**Table:** 

Subject	Engineering
Specific subject area	Hybrid material, Mechanics of composite material, Shear loading, Material properties
Type of data	1. Tables
	2. Figures
Data collection	Data were acquired from Universal Testing Machine (UTM) and the output are load-displacement. Raw data from UTM output are Load in Newton (N) and displacement in millimetres (mm). Additional parameter added from sample measurement such as thickness, width, and length. Material properties that obtained from tensile loading can be transformed to shear stress and it can give information about material strength
Data source location	Data were obtained from the Advanced Manufacturing lab, department of Mechanical and Industrial Engineering, Gadjah Mada University
Data accessibility	https://figshare.com/articles/dataset/Raw_data_of_Arcan_fixture/25046090
Related research article	

## 1 Value of the Data

• The data presented in the current study provide a complete material performance during the shear loading of pure GFRP laminates and hybrid GFRP graphite filler laminates.
• The shear of different loading angle on GFRP laminates from the experiment using a complex Arcan fixture also presented.
• The data of the shear test of GFRP laminates can be used in several fields such as structural applications and automotive industries
• Since load-displacement is a raw data, data processing can be done with different purpose to obtain material properties in term of shear.

## 2 Background

The Arcan fixture is a fixture used to perform shear tests on a specimen. In this case, the specimen is a composite material. The angle of the loading direction of the shear test can be changed and the failure can be analyzed and observed. The Arcan fixture can be easily used by simply positioning the specimen and fixture on the UTM (Universal Testing Machine) machine so that different angles of loading direction can be realized. In the case of in-plane loading, the effect of transverse stress on longitudinal strength is a situation that needs to be considered. The combination of compressive-shear loading is very important in some applications, such as structural profiles (wings or turbine blades) under aerodynamic loads, which generally induce bending (tension and compression) combined with shear. Mechanical property degradation effects will result from the combination of compressive lateral stress and shear with a reduction in fatigue strength and also a change in damage mechanism with respect to tensile loading. [1]. This paper presents a comprehensive dataset that addresses the shear properties of GRP composites when subjected to shear loading using the complex Arcan fixture.

## 3 Data Description

**Fig. 1:**
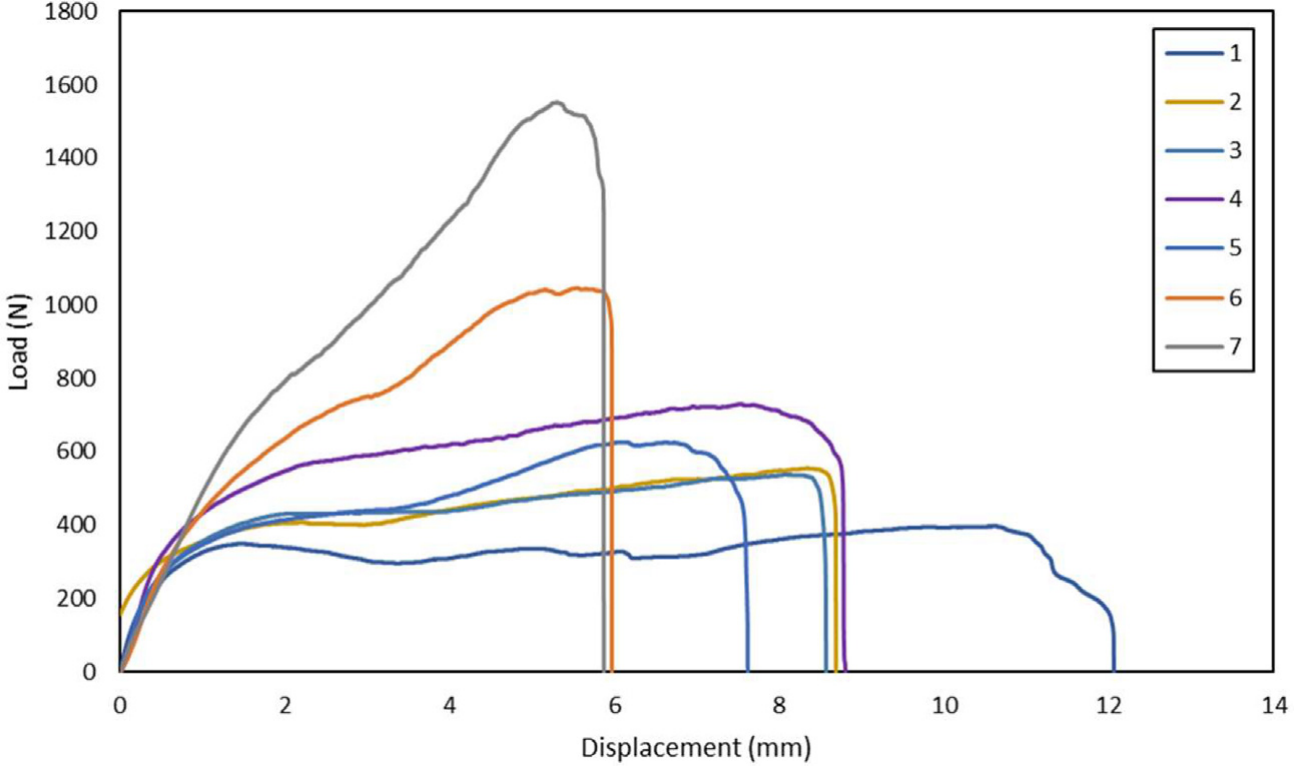
Load-displacement of GFRP laminates shear test in 0°, 10°, 15°, 20°, 30°, 40°, 45° loading angle direction.

**Fig. 2:**
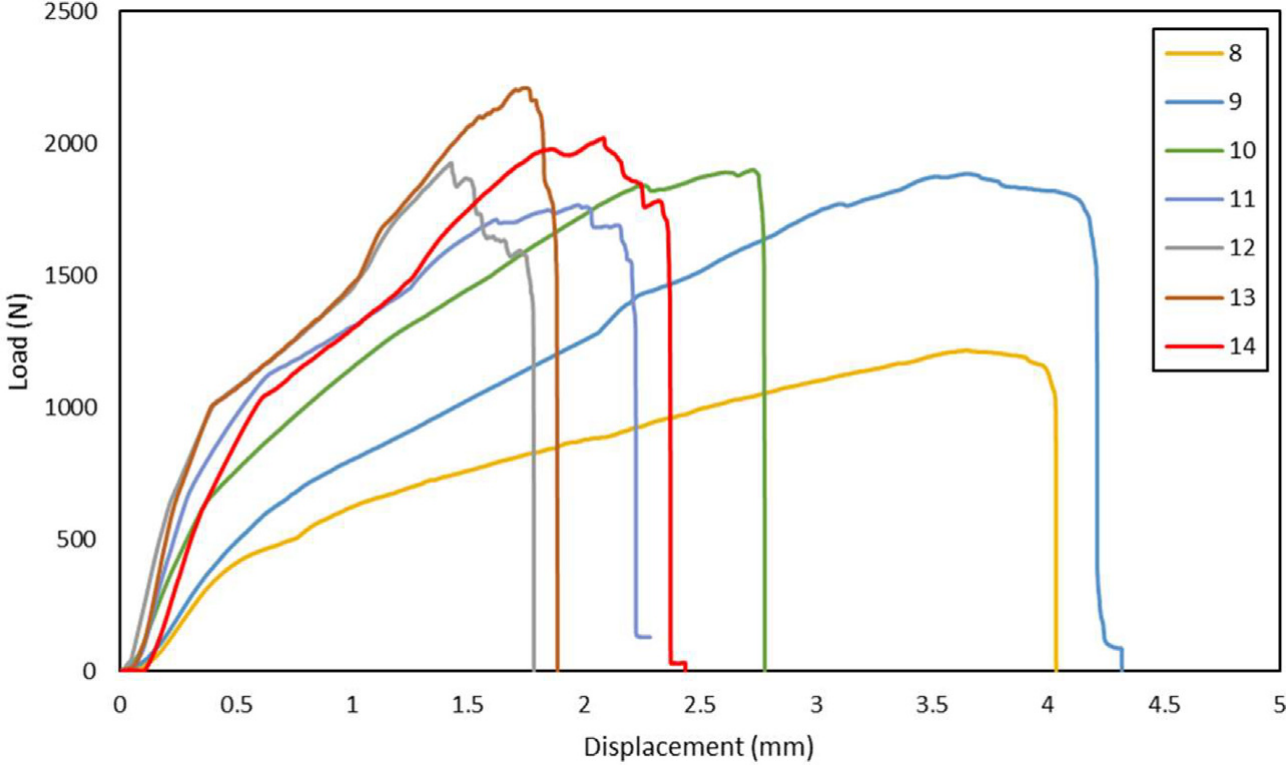
Load-displacement of GFRP laminates shear test in 50°, 60°,70°,75°, 80°, 90°, 105° loading angle direction.

**Fig. 3:**
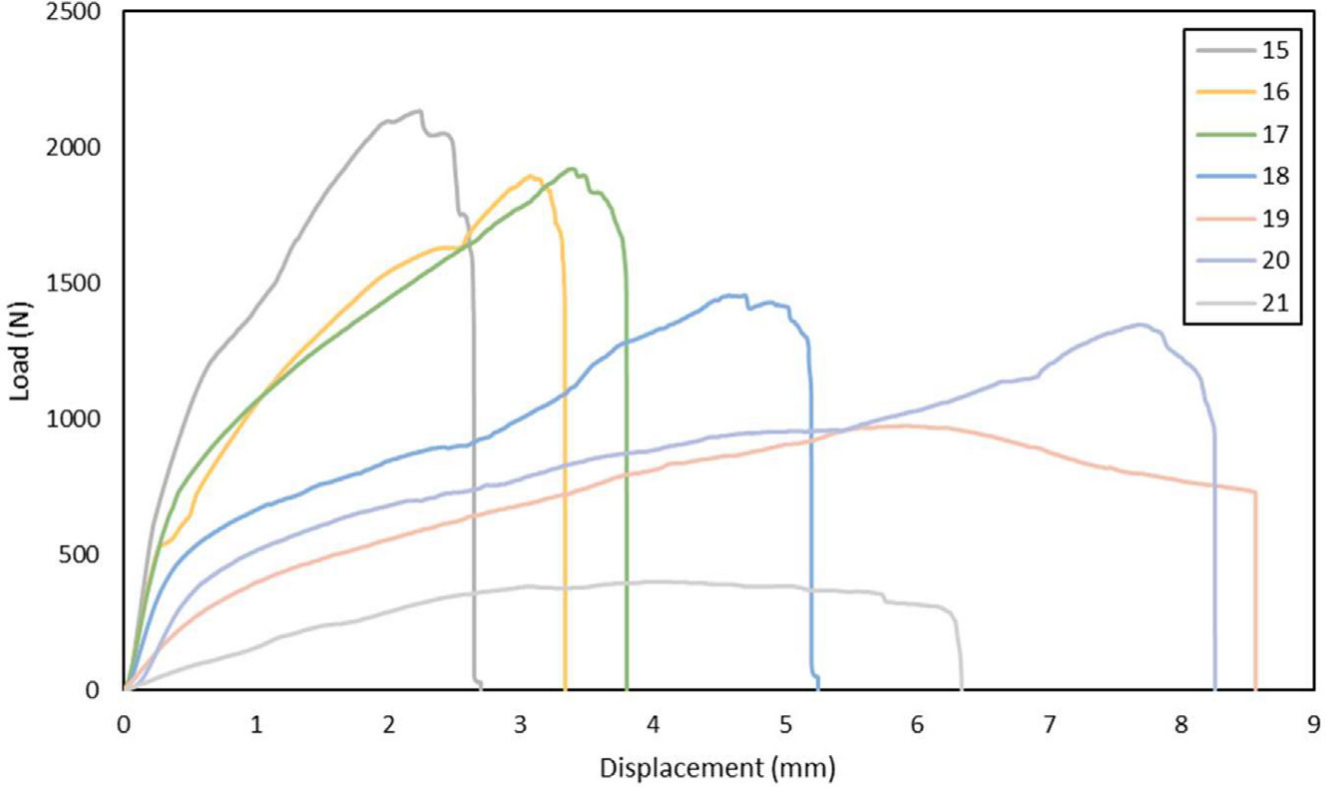
Load-displacement of GFRP laminates shear test in 100°, 110 ^o,^ 120°, 130°, 135°, 140°, 150° loading angle direction.

**Fig. 4:**
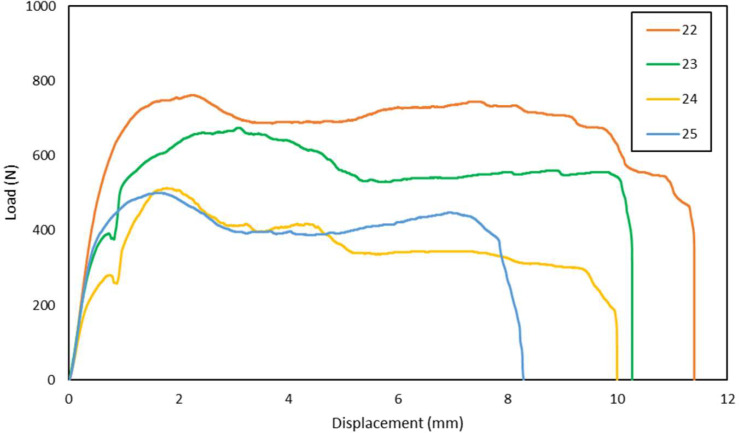
Load-displacement of hybrid GFRP-graphite filler laminates shear test in 0° loading angle direction.

**Fig. 5:**
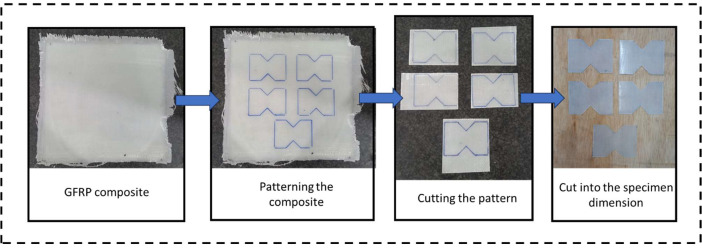
Specimen cutting process.

**Fig. 6:**
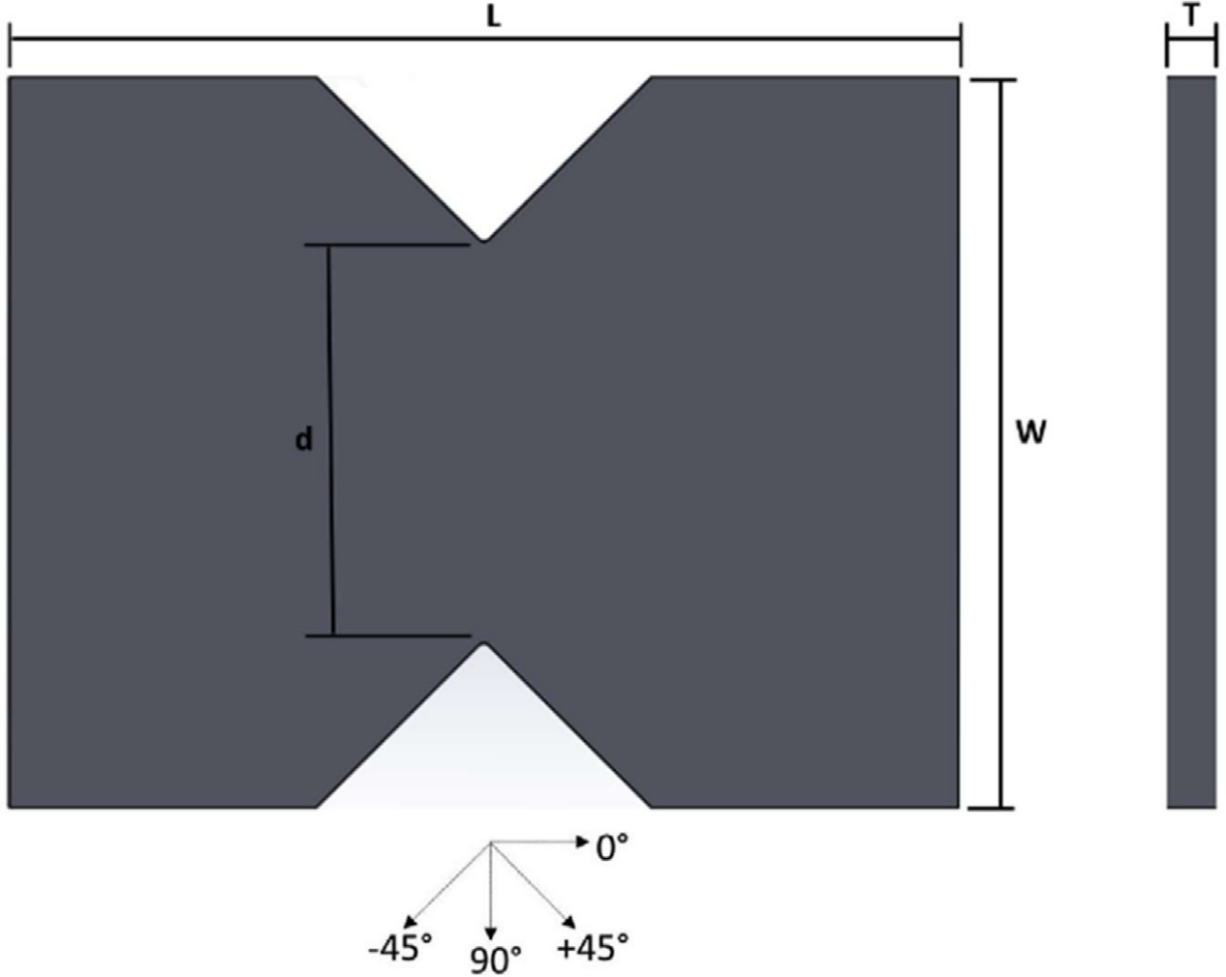
Specimen dimension.

Comprehensive raw data of load-displacement values are available. The data consist of testing pure GFRP laminates specimen and hybrid GFRP graphite filler laminates in terms of shear loading and modifying the loading angle direction can be seen in Table 1. Detailed dimension of all specimens can be seen in Table 2 and the specimen shape is shown in Fig. 6. From one specimen, three point of measurement was taken and calculate the mean. Notch thickness and notch width is the main section to be measured to be included in the equation. Load-displacement of the shear test of pure GFRP laminates is shown in Fig. 1, Fig. 2, Fig. 3, and Fig. 4. The reason we separate it into 4 figures is to clarify the load displacement to be easier to analysed. Each loading angle direction shows a different behavior on its graph. The load-displacement of hybrid GFRP graphite filler laminates can be seen in Fig. 5.

**Table 1:** List of specimens.

No	Composite	Orientation (°)	Loading Angle Direction (°)
1	GFRP	[90/0]_4_	0
2	GFRP	[90/0]_4_	10
3	GFRP	[90/0]_4_	15
4	GFRP	[90/0]_4_	20
5	GFRP	[90/0]_4_	30
6	GFRP	[90/0]_4_	40
7	GFRP	[90/0]_4_	45
8	GFRP	[90/0]_4_	50
9	GFRP	[90/0]_4_	60
10	GFRP	[90/0]_4_	75
11	GFRP	[90/0]_4_	80
12	GFRP	[90/0]_4_	90
13	GFRP	[90/0]_4_	100
14	GFRP	[90/0]_4_	105
15	GFRP	[90/0]_4_	110
16	GFRP	[90/0]_4_	120
17	GFRP	[90/0]_4_	130
18	GFRP	[90/0]_4_	135
19	GFRP	[90/0]_4_	140
20	GFRP	[90/0]_4_	150
21	GFRP + Graphite filler (0.5%)	[90/0]_4_	0
22	GFRP + Graphite filler (1%)	[90/0]_4_	0
23	GFRP + Graphite filler (1.5%)	[90/0]_4_	0
24	GFRP + Graphite filler (2%)	[90/0]_4_	0

**Table 2:** Dimensions of all specimens.

No	Specimen	Notch width (mm)	Notch mean width (mm)	Notch thickness (mm)	Notch mean thickness (mm)	Loading angle direction (°)
1	GFRP	30.98	30.92	0.81	0.80	0
		31.02		0.78		
		30.78		0.81		
2	GFRP	30.66	30.81	0.80	0.80	10
		31.01		0.81		
		30.87		0.80		
3	GFRP	30.65	30.86	0.79	0.79	15
		31.41		0.80		
		30.52		0.80		
4	GFRP	30.59	30.90	0.80	0.79	20
		31.11		0.78		
		31.01		0.81		
5	GFRP	31.21	31.01	0.80	0.80	30
		30.97		0.80		
		30.86		0.81		
6	GFRP	30.45	30.82	0.80	0.79	40
		30.92		0.79		
		31.10		0.79		
7	GFRP	30.93	30.85	0.80	0.80	45
		30.65		0.80		
		30.97		0.81		
8	GFRP	31.25	31.12	0.78	0.79	50
		31.14		0.81		
		30.97		0.80		
9	GFRP	31.23	30.90	0.81	0.79	60
		30.88		0.80		
		30.59		0.79		
10	GFRP	31.21	31.03	0.80	0.80	70
		30.87		0.80		
		31.02		0.80		
11	GFRP	30.65	30.80	0.78	0.79	75
		31.06		0.81		
		30.69		0.80		
12	GFRP	30.97	31.07	0.80	0.80	80
		31.03		0.81		
		31.22		0.80		
13	GFRP	30.94	30.98	0.80	0.79	90
		31.13		0.78		
		30.87		0.81		
14	GFRP	31.12	30.98	0.80	0.80	100
		30.96		0.80		
		30.87		0.81		
15	GFRP	30.45	30.78	0.80	0.79	105
		31.06		0.79		
		30.85		0.79		
16	GFRP	31.07	30.98	0.80	0.80	110
		31.05		0.80		
		30.84		0.81		
17	GFRP	31.09	31.07	0.78	0.78	120
		31.12		0.80		
		31.01		0.78		
18	GFRP	30.96	30.96	0.81	0.80	130
		31.04		0.80		
		30.89		0.80		
19	GFRP	31.05	30.87	0.80	0.80	135
		30.69		0.81		
		30.87		0.80		
20	GFRP	31.02	30.88	0.78	0.79	140
		30.84		0.81		
		30.79		0.80		
21	GFRP	31.20	31.1	0.80	0.80	150
		31.14		0.81		
		30.96		0.80		
22	GFRP + Graphite filler (0.5%)	30.81	30.96	0.79	0.80	0
		31.05		0.80		
		31.02		0.80		
23	GFRP + Graphite filler (1%)	31.06	31.02	0.81	0.80	0
		31.13		0.79		
		30.89		0.80		
24	GFRP + Graphite filler (1.5%)	31.04	30.82	0.80	0.79	0
		30.86		0.78		
		30.56		0.80		
25	GFRP + Graphite filler (2%)	30.95	30.99	0.81	0.80	0
		30.89		0.80		
		31.14		0.80

## 4 Experimental Design, Materials, and Methods

In terms of shear, the experiment should be carried out on an optimum-sized specimens to investigate variations in shear behavior, a fairly uniform and pure shear stress state within the specimen gauge section can only be achieved by a select few test procedures, making shear testing of composite materials a complex process. [3,4]. Composites have been widely used in industry due to their mechanical properties and the composite response in terms of shear stress is a crucial factor to consider. [5]. The interlaminar shear strength (ILSS) of high-modulus continuous fiber-reinforced composite materials depends mostly on the properties of the fiber/matrix interface and the matrix properties. [6,7]. Shear and tensile stress performance will decrease, as notch sensitivity is caused by the redistribution of stress due to progressive damage prior to final failure. [8,9].

The GFRP composite in this study is manufactured using a bi-directional lamina [90/0] consisting of 4 layers of glass fiber, achieving a thickness of approximately 0.8 mm. To ensure consistency in weight and thickness, the composite will be manufactured using the Vacuum Assisted Resin Infusion (VARI) method. The matrix will consist of Epoxy resin Eposchon Bisphenol A (Resin) and Epoxy Hardener EPH 555, mixed gently in a 3:1 ratio. The composite will cure after 24 hours according to the manufacturer's guidelines and will then be unmolded. The dry GFRP will be cut to the approximate dimensions of the ASTM D7078 specimen using a mini grinder, as shown in Fig. 5. The hybrid GFRP graphite filler was also tested using the same ratio of Epoxy resin Eposchon Bisphenol A (Resin) and Epoxy Hardener EPH 555, but with a slightly reduced weight percentage to accommodate the graphite filler. Filler percentages of 0.5%, 1%, 1.5%, and 2% were calculated and added to the mixture.

**Fig. 7:**
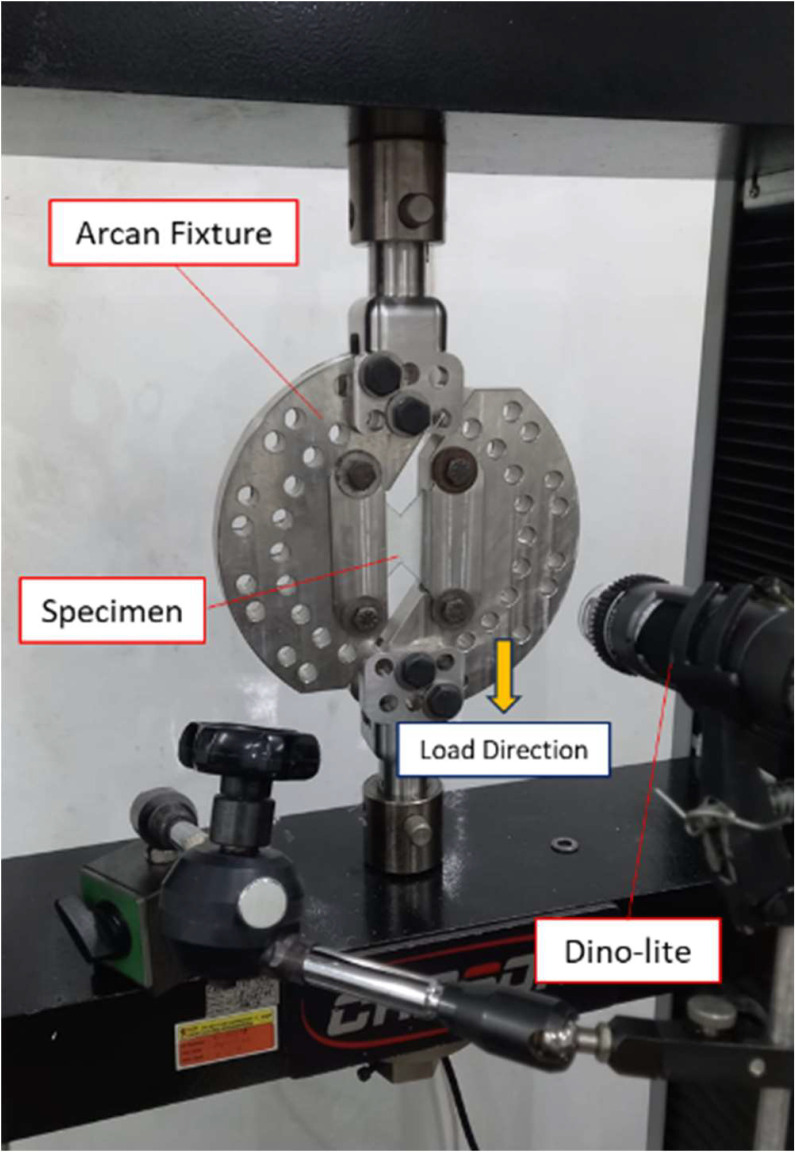
Experimental setup [2].

Shear test was conducted using Carson CRN – 50 Universal Testing Machine (UTM). During the shear test, load-displacement data were recorded automatically until the specimen failed eventually. Along the shear test, Dino-lite 20-200 optical microscope were used to documented the experiment. A complex Arcan fixture is used in this study where the loading angle direction with the angle of 0°, 10°, 15°, 20°, 30°, 40°, 45°, 50°, 60°,70°,75°, 80°, 90°, 105°, 100°, 110 ^o,^ 120°, 130°, 135°,140°,150° can be performed. The butterfly GFRP specimen is clamped in the specimen section in the Arcan fixture. The left side of the specimen is clamped and set to be stationary. Meanwhile, the specimen's right side is clamped and pulled due to the displacement tensile force applied by the fixture. The experimental setup is shown in Fig. 7.

### Note from the experiment

• When cutting the specimen into the geometry dimension, rough cut the composite using a mini grinder and then clean it with a sandpaper. This will give a smaller gap tolerance between the desired dimensions.
• Be careful when using the grinder. Make sure to wear lab gloves and glasses in terms of safety
• When clamping the specimen into the Arcan fixture, do not apply too much torque into the nut and bolt. Or else, it can damage the specimen.
• If the load-displacement graph in the UTM shows an anomaly during the shear test, where the displacement continues but the load stops even when the specimen does not show any fracture, it is likely that there is a slip in the specimen. This means that the test needs to be repeated.
• Keep distance during the shear loading process since the delamination of GFRP may cause injury since it usually forms sharp debris

## Limitations

The dataset has certain constraint. The notch section is become the most influential part on to the influence of shear loading. Angle on its tip can affect the result of the shear strength.

## Ethics Statement

The current work does not involve human subjects, animal experiments, or any data collected from social media platforms.

## CRediT authorship contribution statement

**Ariyana Dwiputra Nugraha:** Conceptualization, Methodology, Investigation, Formal analysis, Software, Data curation, Validation, Visualization, Writing – review & editing. **Daffa Alandro:** Conceptualization, Methodology, Investigation, Formal analysis, Software, Data curation, Validation, Visualization, Writing – review & editing. **Alvin Dio Nugroho:** Writing – original draft, Writing – review & editing. **Eko Supriyanto:** Writing – original draft, Writing – review & editing. **Fefria Tanbar:** Writing – original draft, Writing – review & editing. **Muhammad Akhsin Muflikhun:** Conceptualization, Methodology, Investigation, Formal analysis, Software, Data curation, Validation, Visualization, Writing – review & editing.

## Data Availability

Raw data of Arcan Fixture (Original data) (Figshare).
